# Supplementary figures and images for: Biological Activities and Chemical Profile of Gentiana asclepiadea and Inula helenium Ethanolic Extracts
Source: Molecules. 2022 May 31;27(11):3560. doi: 10.3390/molecules27113560 (PMC9182457; doi:10.3390/molecules27113560)

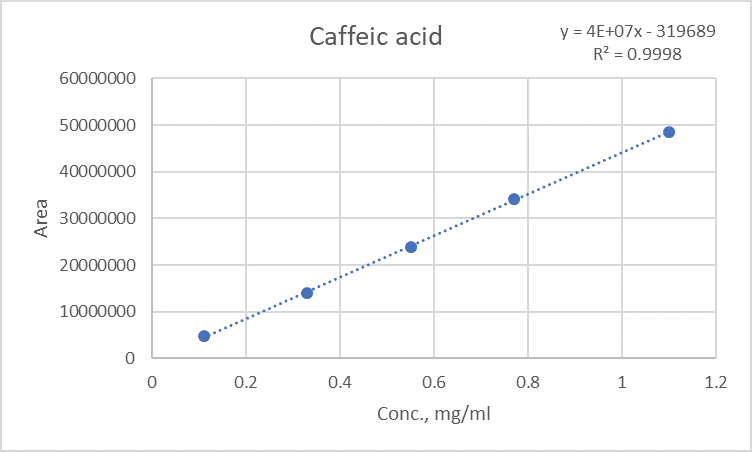

Supplement: Supplementary file 1 [file molecules-27-03560-s001.zip › Figure S1 - The calibration curve of caffeic acid.png]

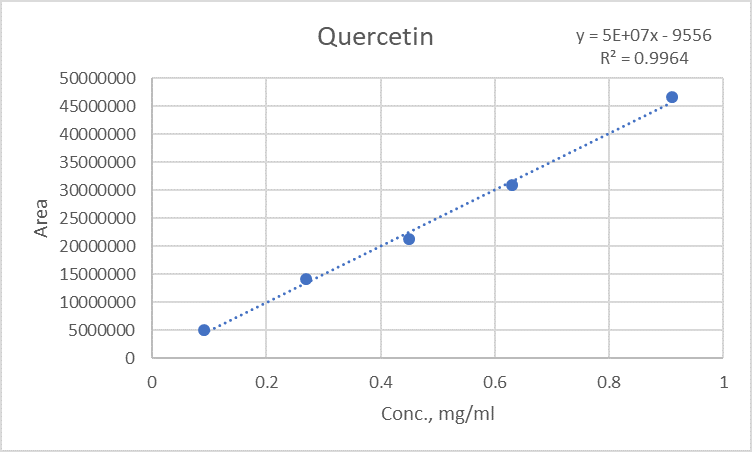

Supplement: Supplementary file 1 [file molecules-27-03560-s001.zip › Figure S10 - The calibration curve of quercetin.png]

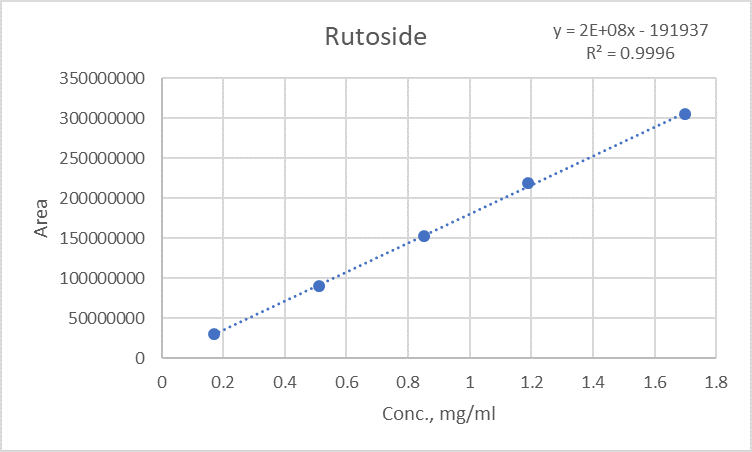

Supplement: Supplementary file 1 [file molecules-27-03560-s001.zip › Figure S11 - The calibration curve of rutoside.png]

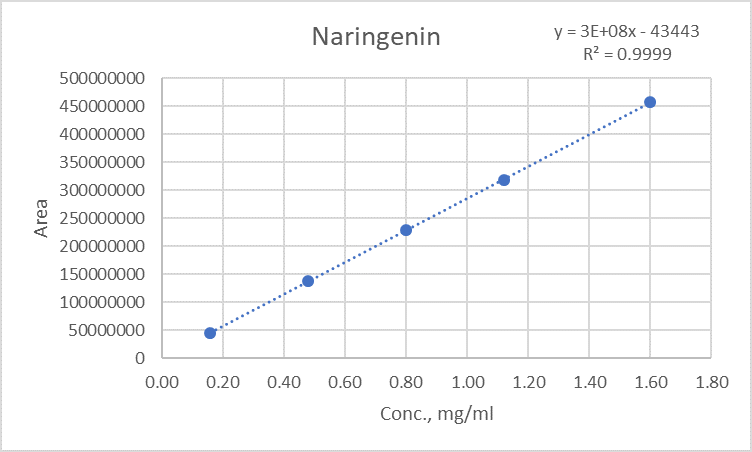

Supplement: Supplementary file 1 [file molecules-27-03560-s001.zip › Figure S12 - The calibration curve of naringenin.png]

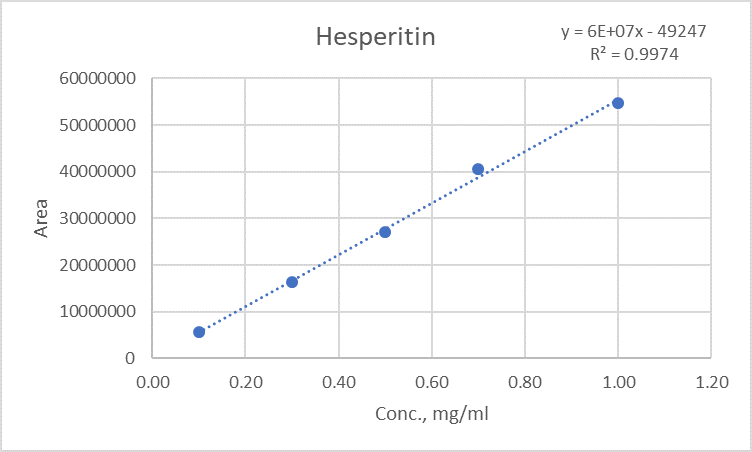

Supplement: Supplementary file 1 [file molecules-27-03560-s001.zip › Figure S13 - The calibration curve of hesperitin.png]

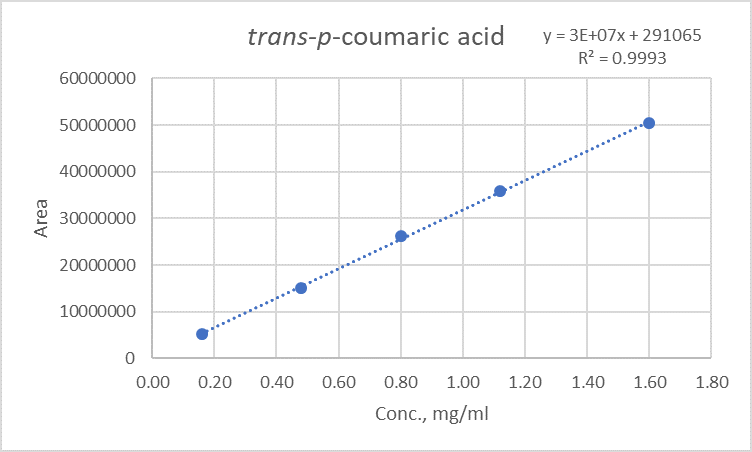

Supplement: Supplementary file 1 [file molecules-27-03560-s001.zip › Figure S2 - The calibration curve of trans-p-coumaric acid.png]

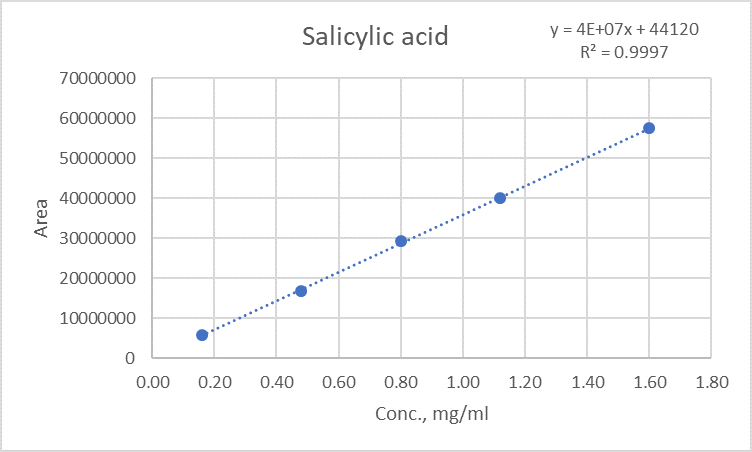

Supplement: Supplementary file 1 [file molecules-27-03560-s001.zip › Figure S3 - The calibration curve of salicylic acid.png]

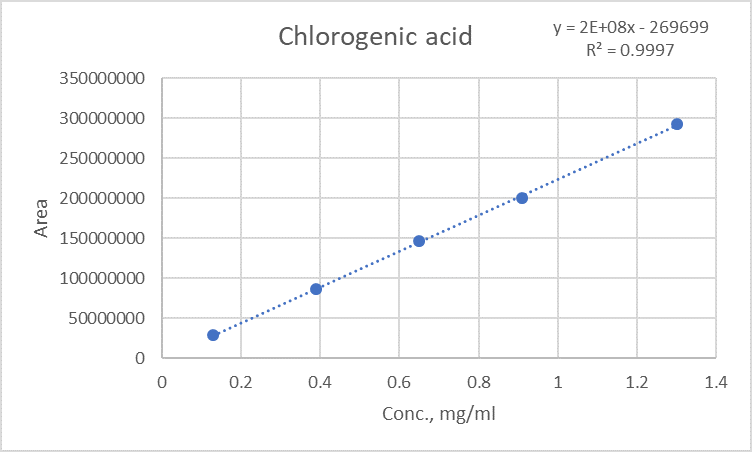

Supplement: Supplementary file 1 [file molecules-27-03560-s001.zip › Figure S4 - The calibration curve of chlorogenic acid.png]

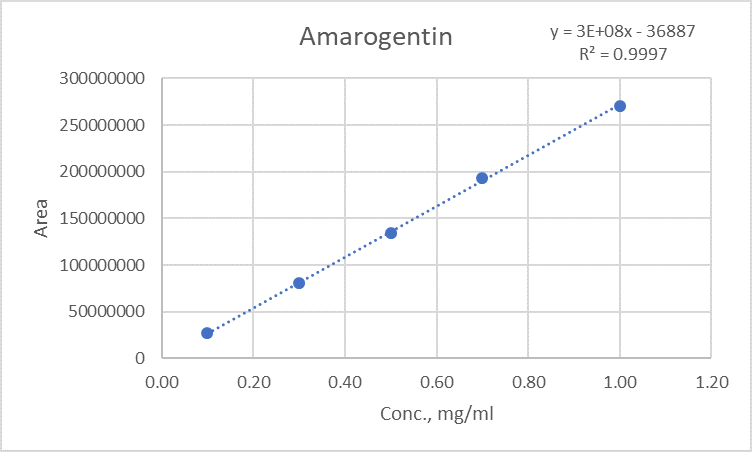

Supplement: Supplementary file 1 [file molecules-27-03560-s001.zip › Figure S5 - The calibration curve of amarogentin.png]

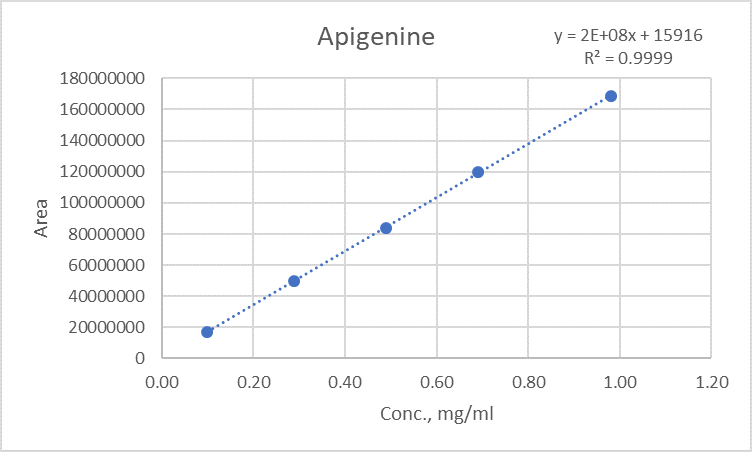

Supplement: Supplementary file 1 [file molecules-27-03560-s001.zip › Figure S6 - The calibration curve of apigenine.png]

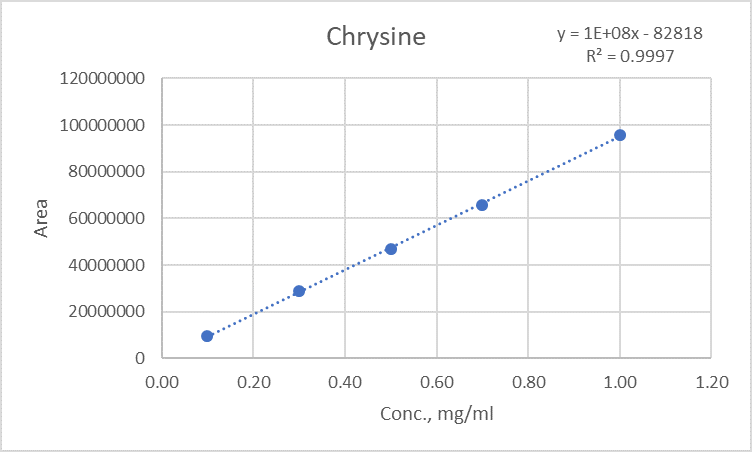

Supplement: Supplementary file 1 [file molecules-27-03560-s001.zip › Figure S7 - The calibration curve of chrysine.png]

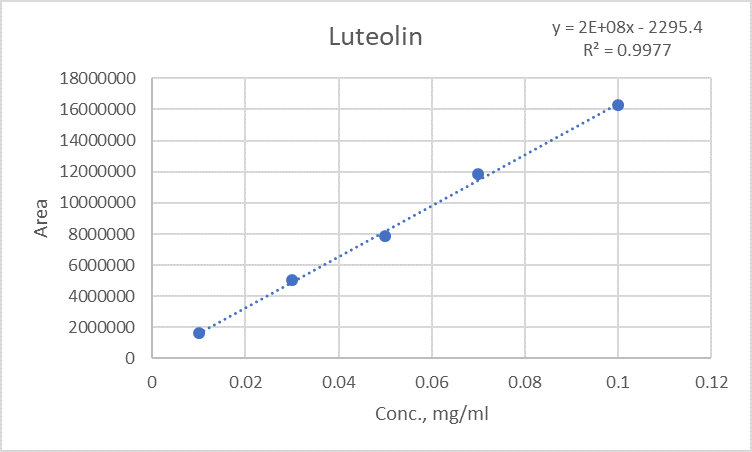

Supplement: Supplementary file 1 [file molecules-27-03560-s001.zip › Figure S8 - The calibration curve of luteolin.png]

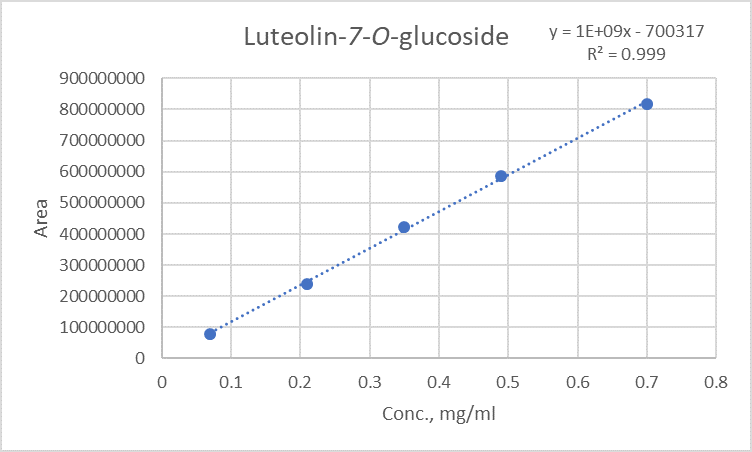

Supplement: Supplementary file 1 [file molecules-27-03560-s001.zip › Figure S9 - The calibration curve of luteolin-7-O-glucoside.png]
